# Admission to hospital for pneumonia and influenza attributable to 2009 pandemic A/H1N1 Influenza in First Nations communities in three provinces of Canada

**DOI:** 10.1186/1471-2458-13-1029

**Published:** 2013-10-30

**Authors:** Michael E Green, Sabrina T Wong, Josée G Lavoie, Jeff Kwong, Leonard MacWilliam, Sandra Peterson, Guoyuan Liu, Alan Katz

**Affiliations:** 1 Departments of Family Medicine and Public Health Sciences, Queen's University, Kingston, Ontario, Canada; 2 Centre for Health Services and Policy Research and Institute for Clinical Evaluative Sciences, Queen’s University, Kingston, Ontario, Canada; 3 School of Nursing and Centre, University of British Columbia, Vancouver, BC, Canada; 4 Centre for Health Services and Policy Research, University of British Columbia, Vancouver, BC, Canada; 5 School of Health Sciences, University of Northern British Columbia, Prince George, BC, Canada; 6 Department of Family and Community Medicine and Dalla Lana School of Public Health, University of Toronto, Toronto, Ontario, Canada; 7 Institute for Clinical Evaluative Sciences, Toronto, Ontario, Canada; 8 University Health Network, Toronto, Ontario, Canada; 9 Public Health Ontario, Toronto, Ontario, Canada; 10 Manitoba Centre for Health Policy, University of Manitoba, Winnipeg, Manitoba, Canada; 11 Departments of Family Medicine and Community Health Sciences, Manitoba Centre for Health Policy, University of Manitoba, Winnipeg, Manitoba, Canada

## Abstract

**Background:**

Early reports of the 2009 A/H1N1 influenza pandemic (pH1N1) indicated that a disproportionate burden of illness fell on First Nations reserve communities. In addition, the impact of the pandemic on different communities may have been influenced by differing provincial policies. We compared hospitalization rates for pneumonia and influenza (P&I) attributable to pH1N1 influenza between residents of First Nations reserve communities and the general population in three Canadian provinces.

**Methods:**

Hospital admissions were geocoded using administrative claims data from three Canadian provincial data centres to identify residents of First Nations communities. Hospitalizations for P&I during both waves of pH1N1 were compared to the same time periods for the four previous years to establish pH1N1-attributable rates.

**Results:**

Residents of First Nations communities were more likely than other residents to have a pH1N1-attributable P&I hospitalization (rate ratio [RR] 2.8-9.1). Hospitalization rates for P&I were also elevated during the baseline period (RR 1.5-2.1) compared to the general population. There was an average increase of 45% over the baseline in P&I admissions for First Nations in all 3 provinces. In contrast, admissions overall increased by approximately 10% or less in British Columbia and Manitoba and by 33% in Ontario. Subgroup analysis showed no additional risk for remote or isolated First Nations compared to other First Nations communities in Ontario or Manitoba, with similar rates noted in Manitoba and a reduction in P&I admissions during the pandemic period in remote and isolated First Nations communities in Ontario.

**Conclusions:**

We found an increased risk for pH1N1-related hospital admissions for First Nations communities in all 3 provinces. Interprovincial differences may be partly explained by differences in age structure and socioeconomic status. We were unable to confirm the assumption that remote communities were at higher risk for pH1N1-associated hospitalizations. The aggressive approach to influenza control in remote and isolated First Nations communities in Ontario may have played a role in limiting the impact of pH1N1 on residents of those communities.

## Background

The 2009 A/H1N1 influenza pandemic (pH1N1) originated in Mexico and quickly spread around the world. The first Canadian cases were reported in April of that year. Early descriptive studies focused on risk factors for severe disease and identified key patterns such as the increased risk for children and pregnant women and younger adults as well as those with chronic conditions [1-3]. It was also assumed that Indigenous^a^ communities in several countries including the United States, Australia, New Zealand and Canada were at increased risk: this assumption was confirmed in later studies [4-8]. As a consequence, public health officials in Canada began to consider specific recommendations for Aboriginal Canadians, which includes First Nations, Inuit, and Métis.

Prior studies in Canada have examined primarily laboratory-confirmed cases [8-11] and have either examined national or provincial rates, but did not compare these across provinces. Internationally, most prior research has also focused on lab-confirmed hospitalized cases. We are not aware of any other Canadian studies examining the population-based impacts across provinces or between Aboriginal communities and the general population. Yet, inequities between Aboriginal and other Canadians persist for virtually every measure of health and social status.

The potential for health and health care disparities among those living on a reserve compared to residents of other communities is higher. A reserve is a small portion of what might have previously been part of a nation’s traditional territory, which is protected by legislation for the use and benefit of a First Nation [12]. Although more than half (51%) of the First Nations population in the provinces of British Columbia, Manitoba, and Ontario live in urban areas [13-15], there continues to be a significant portion of First Nations individuals who live on reserves that are considered rural and remote geographic regions. Further, it has been documented that many First Nations people who live off-reserve travel back to their home reserve to access healthcare [16,17].

This study was undertaken to assess whether there were observed differences in the rates of hospitalizations attributable to pH1N1 in British Columbia, Manitoba, and Ontario between residents of First Nations communities and other residents, and between First Nations in each province.

## Methods

We used administrative data to examine rates of hospitalizations attributed to pH1N1 for those living on First Nations reserves and the rest of the population in British Columbia, Manitoba, and Ontario. Results were aggregated for all First Nations reserve communities in each province. We did not merge the British Columbia, Manitoba, or Ontario datasets because of documented data coding variability.

### Sources of data

The data included files held at Population Health Data British Columbia, the Manitoba Centre for Health Policy, and the Institute for Clinical Evaluative Sciences (ICES) in Ontario. Standardized data, based on every hospital contact, are submitted (including scrambled personal health identifiers, diagnoses, costs, hospitalization, and institutionalization data) as part of a system maintained and controlled by all provincial ministries of health. We used the hospitalization data available through each province’s Discharge Abstract Database (DAD) files and the demographic data available through province-specific datasets (Consolidation file in British Columbia, the Population Registry in Manitoba, and the Registered Persons Database in Ontario) in order to determine sex, age, and location of residence. Socioeconomic quintiles were identified using standard protocols in each province that utilize a combination of census data and location of residence. We also utilized the laboratory influenza testing result files from each province to identify the time periods of interest for waves 1 and 2 of pH1N1 in each province.

### FN identification

In most provinces, current databases are unable to reliably report First Nations identification. Therefore, we created an ecological sample that was assumed to include all British Columbia, Manitoba, and Ontario First Nations living on a reserve. Our unit of interest was residents of First Nations reserve communities. Based on previous work by Lavoie and colleagues we used a combination of 6-digit postal code and First Nations reserve numbers (from Indians and Northern Affairs Canada) to identify all BC, Manitoba, or Ontario residents living on a First Nations reserve [18]. In all three provinces Registered First Nations represent over 90% of the overall on-reserve population [16]. The remainder of the on-reserve population will be the children of one Registered parent and Métis or non-indigenous individuals, all of whom depend on the same services.

Geographic mismatching can be a concern in any rural area, where many small communities may exist within a single postal code. In British Columbia this is of particular concern as postal codes tend to cover larger areas, and First Nations reserves are small. In the case of First Nations, the payer of British Columbia provincial health premiums is the First Nation Inuit Health Branch of Health Canada. Therefore, we were able to use both a proxy for First Nations identification (premium payer) and postal codes where reserves are located to track British Columbia First Nations individuals living on reserve.

### Outcome measures

Since influenza is infrequently confirmed by laboratory tests and the criteria for testing for pH1N1 varied over the study period, and between provinces, we estimated pH1N1-attributable outcomes by applying statistical methods to administrative data. Pneumonia and influenza (P&I) hospitalization (International Classification of Diseases, Tenth Revision [ICD-10] J10-J18) in any diagnostic field was the primary outcome. We examined as secondary outcomes: i) hospitalizations due to pneumonia and influenza as the primary diagnosis; and ii) any respiratory condition (ICD-10 J00-J99) in any diagnostic field. To confirm the specificity of any observed differences, we examined motor vehicle collisions (ICD-10 V01-V79) as a condition where pH1N1 was not expected to have an impact.

### Data analysis

Data analyses were conducted within each province. Frequent communication during the analysis stage ensured consistency in analyses despite slight variations inherent in the health administrative data. To estimate pH1N1-attributable outcomes, we first defined periods of pH1N1 activity (i.e., pandemic waves 1 and 2, which were approximately April-June and October-November 2009) using provincial laboratory data. We then determined event rates during these periods in 2009 and compared them to event rates during the same periods in the previous five years (2004 to 2008). The rate during the previous years served as the expected baseline for these outcomes, controlling for inherent differences between provinces and between First Nations communities and the general population in terms of health services utilization, public health prevention and control strategies, and influenza disease rates. pH1N1-attributable event rates were calculated as the difference between the pH1N1 waves in 2009 and the baseline periods in previous years.

We conducted analyses that included crude and age standardized rates and rate ratios. Rates of admission were first determined for each week then aggregated to generate average weekly rates for the time periods of interest. For Ontario and Manitoba we also used the First Nations and Inuit Health rurality designation to determine if there were differences between Remote and Isolated First Nations reserve communities and those rated as Semi-Isolated and Non-Isolated. In Ontario, the locations and categories of each community in our dataset were compared to the information in the First Nations Community Profiles on the Aboriginal Affairs and Northern Development Canada website to ensure they were accurate. One of the investigators (MG) has content expertise in this area and was able to resolve any conflicts identified during this processes. In Manitoba this information was already available and verified from previous work by one of the investigators(JL). This was not possible in British Columbia due to the nature of the dataset obtained from Population Health Data British Columbia, where aggregation beyond the community level had already been completed prior to release of the data set, making it impossible to use this approach. We used a rate ratio approach as the relatively low event counts in the First Nations communities precluded the meaningful use of regression models to adjust for confounding variables. We used SAS 8.0/9.2 for data analysis depending on the version in use at each data centre [19].

### Ethics and partnerships

This project was conducted in keeping with the Tri-Council Policy Statement on Research involving Aboriginal Peoples [20]. As we were using provincial-level datasets, guidance and input into the study was provided by provincial-level First Nations organizations. Research agreements were implemented between the research team and Chiefs of Ontario, the Assembly of Manitoba Chiefs, and the British Columbia First Nations Health Council. The roles of the partners included review and approval of the application for funding, the methodology used for identifying First Nations communities, review of preliminary results and review, and approval of the final manuscript.All procedures were approved by ethics boards at the Univeristy of British Columbia, University of Manitoba, and Queen’s University. Data access requests were approved by Population Data British Columbia, The Health Information Privacy Committee in Manitoba, and the Institute for Clinical Evaluative Sciences.

## Results

There were key demographic differences between First Nations reserve communities and other residents in each of the three provinces (Table 1). Residents of First Nations reserves were more likely to be younger, have lower socioeconomic status and less likely to reside in an urban area (as would be expected given the location of most reserves). The age and SES differences were greatest in Manitoba and smallest in British Columbia. Ontario had several reserve communities located in urban census metropolitan areas, while there were none in Manitoba. British Columbia data were not available for this particular analysis.

**Table 1:** Sociodemographic characteristics of First Nations Reserves and Other Residents in British Columbia, Manitoba, and Ontario, 2009.

	**British Columbia**		**Manitoba**		**Ontario**	
	**First Nations Reserves**	**Other residents**	**First Nations Reserves**	**Other residents**	**First Nations Reserves**	**Other residents**
Age <16 years	26.9%	17.2%	35.2%	19.6%	32.4%	18.4%
Age >65 years	6.1%	13.6%	5.5%	13.6%	7.06%	13.05%
% Female	48.8%	50.6%	49.1%	50.8%	50.4%	50.1%
% Urban	NA	NA	0.02%	65.1%	12.2%	76.8%
SES Quintiles						
1	45.1%	20.1%	61.9%	16.8%	49.1%	20.8%
2	16.5%	20.1%	17.1%	19.9%	11.8%	20.1%
3	14.5%	20.2%	16.2%	20.2%	7.2%	19.6%
4	13.6%	20.0%	2.7%	21.0%	14.0%	19.8%
5	10.3%	19.6%	1.9%	21.0%	17.9%	19.7%

Urban = Census Metropolitan Area (>100,000).

SES Quintiles excluding missing results.

NA = Not available.

**Figure 1:**
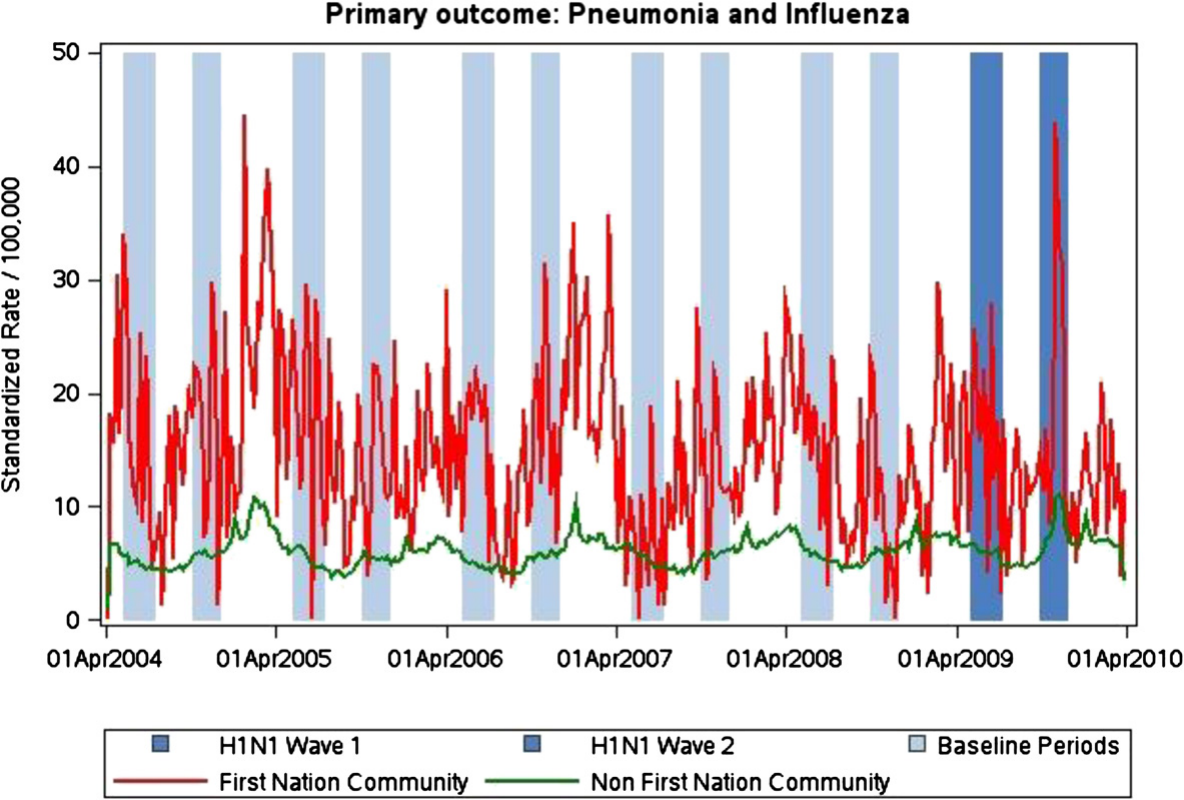
**Weekly hospitalizations rates for pneumonia and influenza in Ontario.** Ontario – standardized rates per 100,000 population per week, April 2004-April 2012. Vertical bars represent waves 1 and 2 of H1N1 and corresponding control periods in earlier years.

Figure 1 presents the weekly hospitalization rates for P&I in one province (Ontario) from April 2004 to April 2010. Hospitalization rates for P&I were higher in First Nations reserves than in other communities throughout this period. The influence of seasonal influenza on admission rates is clearly evident, as is the spike in admissions due to wave 2 of pH1N1.

The remainder of the results are limited to the pandemic periods noted in Figure 1 and the corresponding control periods (same weeks) in each of the prior five years. Table 2 presents the results for our primary outcome. The differences between age-adjusted and crude rates did not significantly influence the results of our primary outcome so we have presented only the crude rates. For both the baseline period and during pH1N1 waves, the rates of admission were highest in Manitoba. All provinces had higher rates of admission for First Nations reserve communities during the baseline period (RR 1.5-2.1). pH1N1-attributable admissions were higher for First Nations reserve communities (RR 2.8-9.1). In all three provinces there was a similar increase (44-46%) in P&I admissions among First Nations reserve communities. In contrast, there was little change in the admission rates for the general population in either British Columbia (8%) or Manitoba (11%), but a 33% increase in Ontario. The “ratio of ratios” indicates the degree by which the proportional increase in P&I related hospitalizations for residents of First Nations Reserve communities was greater than that experienced by the general population after taking into account expected differences based on historical data. This showed the increase in hospitalization rates for residents of First Nations Reserve communities to be 35% higher in BC, 31% higher in Manitoba and 8% higher in Ontario.

**Table 2:** Pneumonia and Influenza Hospitalizations (Crude Rate per 100,000 per week)

	**2009**	**2004-08**	**H1N1 Attributable**	**Rate Ratio: 2009:2004-08**
British Columbia				
First Nations Reserves	13.77	9.48	4.29	1.45
Other Residents	6.65	6.18	0.47	1.08
Rate Ratio:	2.07	1.53	9.13	1.35*
First Nations:Other				
Manitoba				
First Nations Reserves	26.84	18.44	8.40	1.46
Other Residents	11.21	10.07	1.14	1.11
Rate Ratio:	2.39	1.83	7.36	1.31*
First Nations:Other				
Ontario				
First Nations Reserves	16.84	11.74	5.10	1.44
Other Residents	7.35	5.54	1.81	1.33
Rate Ratio:	2.29	2.12	2.81	1.08*
First Nations:Other				

Crude rates of hospitalization for pneumonia or influenza (any diagnostic field) per 100,000 population per week. 2009 represents the average combined rate of both waves of H1N1 and 2004-08 the average rate during the same weeks in each of the prior 5 years. *Indicates the “ratio of ratios” which represents the degree to which the rate of P&I admissions increased relative to the general population after taking into account expected differences in rates based on the rates of hospitalization for P&I in 2004-2009.

Our additional sensitivity analyses showed that when P&I was used as the primary diagnosis, we found similar results to our primary outcome (data not shown). For all respiratory hospitalizations an increase was also noted, but with the degree of this somewhat tempered (increases of approximately 15% rather than 44% in Ontario for example) as would be expected by the inclusion of other non-infectious respiratory admissions in this outcome (data not shown). Sensitivity analyses also showed that there was no temporal relationship noted between motor vehicle collision rates and pH1N1, although motor vehicle collision rates for residents of First Nations reserve communities were also noted to be twice that of the general population (data not shown).

For Manitoba and Ontario, we were also able to compare remote and isolated First Nations reserve communities to those that are not remote or isolated (Table 3). In Manitoba the rates of admission overall and the pH1N1-attributable rates were similar for both groups. In Ontario, there were fewer P&I admissions in remote and isolated communities and there was actually a decrease in admissions between the baseline period and 2009 for these communities. In contrast, there was a 58% increase in admissions for other First Nations reserve communities.

**Table 3:** Pneumonia and Influenza Hospitalizations by Geographic Location – First Nations Communities in Manitoba and Ontario only (Crude rates per 100,000 per week)

	**2009**	**2004-08**	**H1N1 Attributable**	**Rate Ratio: 2009:2004-08**
Manitoba First Nations Communities				
Remote/Isolated	27.69	18.29	9.40	1.51
Other	25.71	18.64	7.07	1.38
Rate Ratio	1.077	0.98	1.32	1.10*
RI:Other				
Ontario First Nations Communities				
Remote/Isolated	7.05	7.91	−0.88	0.89
Other	21.47	13.56	7.91	1.58
Rate Ratio	0.33	0.58	−0.11	0.56*
RI:Other				

Crude rates of hospitalization for pneumonia or influenza (any diagnostic field) per 100,000 population per week. 2009 represents the average combined rate of both waves of H1N1 and 2004-08 the average rate during the same weeks in each of the prior 5 years. *Indicates the “ratio of ratios” which represents the degree to which the rate of P&I admissions in remote or isolated FN communities increased (or decreased) relative to the rates in other FN communities after taking into account expected differences in rates based on rates of hospitalization of P&I in 2004-2009.

British Columbia data was not included as the nature of the data set did not permit this particular subgroup analysis.

### Discussion

This study confirms that at a population level, there was a greater risk of pH1N1-attributable hospitalization (RR = 2.8-9.1) for First Nations reserve communities than the remainder of the population. These health disparities are consistent across BC, Manitoba, and Ontario, and consistent with other studies of hospitalized or laboratory-confirmed cases in North America, Australia, and New Zealand [3-11,21]. For example, Campbell et al. found that the likelihood of hospital admissions for lab confirmed pH1N1 in Canada was 5-7 times greater for those with Aboriginal status compared to the general population [10]. LaRuche et al. compared hospitalization rates for pH1N1 across several countries and found that the relative risk for Indigenous populuations to be between 3.0-7.7 times higher than the general population [4]. They also noted elevated mortality (RR 3.4-5.3) [4], as did Castrodale et al. (RR2.9-5.6) in the United States [5]. Wilson et al. also reported increased mortality for the Maori in New Zealand (RR 2.0-7.2), and further note that this pattern has been consistently observed in all the major pandemics documented since 1918 [21].

We also found elevated baseline risks of P&I hospitalization (RR = 1.5-2.1) variability in both the baseline and pH1N1-attributable hospitalization rates between provinces. Some of this variability may be explained by demographic differences between FNs reserve communities in each province. Manitoba, which had the highest rates, also has the youngest and poorest First Nations reserve population. This would be in keeping with other studies, which have shown a convincing association between lower SES and increased risk of adverse outcomes from H1N1 influenza [22,23]. Once baseline differences are accounted for, the relative increase in risk of hospitalization for pH1N1 for those living on a First Nations reserve was similar across provinces at about 45% (44-46%) compared to prior years. In Ontario, where pH1N1-related hospitalizations mostly occurred during the second wave, there was also a significant impact in the general population, while in BC and Manitoba the impact on the general population was minimal. This would be consistent with a prior study of laboratory-confirmed pH1N1 hospitalizations that found the second wave to have much higher admission rates [11].

Guidelines identified remote or isolated First Nations as being at particularly increased potential risk and in some provinces such as Ontario, more aggressive pandemic control strategies (such as more liberal use of antiviral medication, expanded indications for mask usage and increased consideration of cancellation of mass gatherings) were implemented [24-27]. Our data did not show increased risk for these communities compared to other First Nations reserve communities at baseline, with admission rates being essential identical in Manitoba and actually lower in Ontario. This may be due in part to the types of facilities and healthcare services available locally in these communities as more isolated communities often receive higher levels of services on site. During pH1N1 in Manitoba there was only a slightly higher rate of pH1N1-attributable admission in remote/isolated vs. other First Nations community types (RR = 1.51 vs. RR = 1.38). In Ontario, there was actually a decrease in hospitalizations in remote/isolated First Nations reserve communities during pH1N1, with all of the increase in admissions coming from the other community types. This could be related to the more aggressive approach taken to control and treatment of pH1N1 in these communities or other factors. It should be noted that as pH1N1 was primarily a second wave event in Ontario, the experience of other jurisdictions with the first wave of pH1N1 was taken into consideration during the development and implementation of clinical and public health guidelines in that province. Future guidelines should consider all First Nations communities to be at elevated risk and also address the needs of those less isolated communities that have limited access to on reserve healthcare services.

### Limitations

A number of limitations to the study should be taken into consideration. The first is that we are using pH1N1-attributable rather than laboratory-confirmed hospitalizations as an outcome. We feel this is reasonable because not all patients would necessarily have been tested, but this does potentially lead to incorrect attribution of cases. We did not adjust for a number of known risk factors such chronic diseases, pregnancy, or age group. Moreover, there are other risk factors that are not measurable in administrative data (e.g., access to safe drinking water or overcrowding) [28]. However, our analyses included close to the entire population in each province and age standardization did not have a major impact on our conclusions. The time periods for observation were based on provincial level laboratory data, which could be confounding if the waves of H1N1 on First Nations reserves occurred at different times from the general population. Finally, it is likely that there was some misattribution due to imperfect matching of First Nation reserve boundaries with postal code boundaries. In BC, using a combination of postal code and premium payer to identify First Nations may result in the inclusion of a small number of First Nations living outside but close to the reserve. We believe their access to health services compares to that of the First Nations on-reserve population living adjacent to them.

## Conclusions

The findings reported here are unique in that analyses were carried out across three provinces using their health administrative data to shed light on the impact of pH1N1 among First Nations reserve communities. Findings provide empirical population level evidence of both an absolute and relative increase in risk for pH1N1-attributable hospitalizations for First Nations reserves relative to the general population. The increased baseline risk suggests that this risk is elevated in general and not specific to pH1N1.

The interprovincial variations could indicate that SES, age, and other differences for which we do not have regular sources of data (e.g., housing status) are important mediators of these differences. Unlike what is generally assumed, increasing remoteness of the community was not an important risk factor. Outcomes were not related to where the communities are situated, but rather to the care provided. Future studies should examine more closely the influence of the types of health services delivered locally. Still, the aggressive use of antiviral medications in remote and isolated communities in Ontario may have contributed to the reduced impact of the pandemic in those areas.

## Endnote

^a^Throughout this paper, the term Aboriginal will be used when statements apply to First Nations living on- and off-reserve, Inuit, and Métis. Elsewhere, the terms First Nations, Inuit, and Métis will be used when specific to each of these populations. Finally, the term Indigenous will only be used when speaking in international terms.

## Competing interests

MG serves as a medical officer for First Nations and Inuit Health Branch, Ontario Region and participated in the management of the H1N1 pandemic on First Nations reserves in Ontario.

## Author’s contributions

MG, SW, AK, JL and JK designed the study and contributed to all aspects including the development of partnerships, acquisition of data, analysis and interpretation of results. LM, SP and GL worked directly on the analysis of the administrative data. All authors contributed to the discussion and review of the manuscript. All authors read and approved the final manuscript.

## Pre-publication history

The pre-publication history for this paper can be accessed here:

http://www.biomedcentral.com/1471-2458/13/1029/prepub
